# An Innovative Framework for Delivering Psychotherapy to Patients With Treatment-Resistant Posttraumatic Stress Disorder: Rationale for Interactive Motion-Assisted Therapy

**DOI:** 10.3389/fpsyt.2018.00176

**Published:** 2018-05-04

**Authors:** Marieke J. van Gelderen, Mirjam J. Nijdam, Eric Vermetten

**Affiliations:** ^1^Foundation Centrum'45, Arq Psychotrauma Expert Groep, Diemen, Netherlands; ^2^Department of Psychiatry, Leiden University Medical Center, Leiden, Netherlands; ^3^Department of Psychiatry, Academic Medical Center at the University of Amsterdam, Amsterdam, Netherlands; ^4^Military Mental Health-Research, Ministry of Defence, Utrecht, Netherlands

**Keywords:** PTSD, treatment, veterans, treatment resistance, innovation, virtual reality, physical activity, reconsolidation

## Abstract

Despite an array of evidence-based psychological treatments for patients with a posttraumatic stress disorder (PTSD), a majority of patients do not fully benefit from the potential of these therapies. In veterans with PTSD, up to two-thirds retain their diagnosis after psychotherapy and often their disorder is treatment-resistant, which calls for improvement of therapeutic approaches for this population. One of the factors hypothesized to underlie low response in PTSD treatment is high behavioral and cognitive avoidance to traumatic reminders. In the current paper we explore if a combination of personalized virtual reality, multi-sensory input, and walking during exposure can enhance treatment engagement, overcome avoidance, and thereby optimize treatment effectiveness. Virtual reality holds potential to increase presence and in-session attention and to facilitate memory retrieval. Multi-sensory input such as pictures and music can personalize this experience. Evidence for the positive effect of physical activity on fear extinction and associative thinking, as well as embodied cognition theories, provide a rationale for decreased avoidance by literally approaching cues of the traumatic memories. A dual-attention task further facilitates new learning and reconsolidation. These strategies have been combined in an innovative framework for trauma-focused psychotherapy, named Multi-modular Motion-assisted Memory Desensitization and Reconsolidation (3MDR). In this innovative treatment the therapeutic setting is changed from the face-to-face sedentary position to a side-by-side activating context in which patients walk toward trauma-related images in a virtual environment. The framework of 3MDR has been designed as a boost for patients with treatment-resistant PTSD, which is illustrated by three case examples. The intervention is discussed in context of other advancements in treatment for treatment-resistant PTSD. Novel elements of this approach are activation, personalization and empowerment. While developed for veterans with PTSD who do not optimally respond to standardized treatments, this innovative framework holds potential to also be used for other patient populations and earlier stages of treatment for patients with PTSD.

## Background

For the treatment of patients with a chronic Posttraumatic Stress Disorder (PTSD) several effective psychological treatments are available, of which Trauma-Focused Cognitive Behavioral Therapy and Eye Movement Desensitization and Reprocessing (EMDR) have been investigated most extensively (1, 2). However, meta-analytic studies show that the majority of patients still suffer from substantial residual symptoms posttreatment and that certain patient populations benefit less from the potential of these treatments than others (2–4). One group of patients that have consistently been found to insufficiently respond to evidence-based treatments for PTSD are veterans (2, 3, 5). Trials considering the efficacy of these treatments for veterans generally report a pre- to posttreatment improvement, however approximately two-thirds of the veterans retain their PTSD diagnosis (6). Furthermore, in clinical practice drop-out rates for veterans with PTSD are high, up to 78% (7). Recent evidence from a review on treatment-resistant PTSD (TR-PTSD) suggested that veterans are at high risk for their PTSD to be an unremitting illness (8).

In a meta-regression analysis on predictors for treatment outcome of veterans with PTSD Haagen et al. (9) found the number of trauma-focused therapy sessions, and not the total number of psychotherapy sessions, to predict treatment outcome. Additionally, Jaycox et al. (10) showed that poor emotional engagement during PTSD treatment hinders positive outcome and in a sample of patients with chronic PTSD irregular treatment attendance was found to predict whether a patient benefits from treatment (11). A factor that we hypothesize to underlie these predictors is high avoidance as this would lead to patients being more prone to not show up for treatment sessions, dropping out of treatment, not (fully) participating in exposure therapy, and circumventing trauma-related topics during therapy. Avoidance, in such ways, potentially hinders full treatment engagement and may lead to negative treatment outcome. In line with these thoughts, Badour et al. (12) performed a study in 1073 military veterans and found that avoidance at intake predicted PTSD symptom severity at discharge. Salcioglu et al. (13) found that lack of change in behavioral avoidance symptoms early in treatment was associated with a lack of change in other PTSD symptoms.

These alarming signals have resulted in our ambition, as well of those of policy-makers and other researchers, to attempt to rigorously explore novel out-of-the-box approaches for TR-PTSD patient populations, and more specifically for veterans with TR-PTSD [e.g., (7, 14)]. In the current paper we outline the rationale for an innovative intervention for this patient population, which has the primary objective to increase engagement and decrease cognitive and behavioral avoidance during treatment, because we believe this to be essential for patients to ultimately benefit from treatment. The aim of this paper is to first discuss innovations and augmentative strategies which show potential for a therapeutic approach in which this objective is achieved. In doing so we review the current status of research on virtual reality, multi-sensory inputs and movement in relation to PTSD treatment. We discuss how these components can be combined into an innovative framework for trauma-focused psychotherapy and illustrate its application with three case examples. Strengths and limitations of the novel intervention in the context of other new interventions and innovations for TR-PTSD are discussed. Finally, ongoing research on the efficacy of this new approach and possible future applications of this intervention are presented.

## Rationale for an innovative framework for treatment of PTSD

### The promise of virtual reality

While Virtual Reality (VR) as an augmentation strategy in treatment of mental health disorders was introduced only little over a decade ago with the treatment of specific anxiety disorders, it is now adopted in the treatment of PTSD as well. In phobias, VR allows for a direct graded exposure of the patient to the feared stimuli, such as spiders (15, 16). This has contributed to development of Virtual Reality Exposure Therapy (VRET), in which patients with PTSD can be exposed to (elements of) their traumatic event, with the aim of activating and processing the traumatic memory trace of this event (17). In doing so a central aspect is presence, which refers to the degree to which users engage in the environment and perceive it as being real (18). This is determined by levels of immersion and in-session attention (17). In most VRET research immersion is defined as the degree to which the technical aspects of the VR application allow for a realistic experience (17). In gaming research (19) this type of immersion is referred to as *spatial immersion*. Three other types of immersion are distinguished: *emotional immersion* (players become invested in a story), *sensory motoric immersion* (players perform a motoric task with success) and *cognitive immersion* (players perform a challenging cognitive task with success). VRET aims to immerse patients in environments that strongly resemble their traumatic experiences [spatial immersion; (17)] and let them interact with these environments, thereby leading to higher in-session attention (20). Because of the hypothesized decreased cognitive and behavioral avoidance, patients may more easily retrieve, confront and process their traumatic memories. Since immersion is essential to increase presence and thereby therapeutic efficacy, explicitly addressing other types of immersion during therapy could provide a viable augmentation strategy as well.

Two systematic reviews on the efficacy of VRET for PTSD (21, 22) found this approach to treatment to be as efficacious as traditional imaginal exposure therapies [see Table 1 for an overview of sample investigated, effect sizes, and potential confounders]. However, the most recent review (22) included a large variety of treatment protocols and the treatment effect might therefore be under- or overestimated. The 12 studies included in the review differed in technological devices that were used to administer VR, type of environments used, inclusion of additional sessions of standard Prolonged Exposure (PE) or cognitive restructuring sessions and the amount of sessions applied (between 4 and 20). An issue of interest in the application of the VRET is the type of device used, because it may have implications for the therapeutic process. In VRET, a variety of technological devices can be employed with the two most widely used being a Head Mounted Display (HMD) or a Cave Automatic Virtual Environment (CAVE). Although the two have not yet been compared in treatment of PTSD, some differences are clear. A CAVE consists of screens surrounding the patients, which allows for several people to be in the same environment at the same time and prevents isolating a patient from the outer world (which could evoke dissociation). The HMD on the other hand is thought to more fully immerse patients in the environment. However, these assumptions have not yet been tested, neither has the optimal level of immersion been studied.

**Table 1:** Outcomes of systematic reviews and meta-analysis on VRET and exercise.

**Subject Authors**	**Study**	**Sample (size)**	**Outcome and effect size**	**Potential bias**
**VRET**				
(22)	Systematic review	PTSD patients (*n* = 157), including veterans, victims of criminal violence, survivors of 9-11 terrorist attacks.	In 3 out of 3 studies comparing VRET to a waitlist control group, symptoms improved in VRET group only. In 6 out of 7 studies comparing VRET to an active treatment, PTSD symptoms improved but did not outperform traditional therapies. The other study found VRET to outperform IE. No effect size reported.	- Small sample sizes; largest sample size was 22. - High risk of bias in most studies due to lack of blinding. Some studies did not randomize patients. - High variety of protocols used.
(21)	Systematic review	PTSD patients (*n* = 139), including veterans, victims of criminal violence, survivors of 9–11 terrorist attacks.	VRET improved PTSD symptoms in all studies. In 1 study VRET outperformed a minimal attention control group. No effect size reported.	- Small sample sizes; largest sample size was 36. - High risk of bias in most studies due to lack of blinding. Some studies did not randomize patients. - High variety of protocols used.
(15)	Meta-analysis	Anxiety disorder patients (*n* = 397).	VRET improved PTSD symptoms; Cohen's *d* = 1.11. PTSD outperformed other exposure therapies; Cohen's *d* = 0.35.	- Over-representation of specific phobias (fear of flying and acrophobia).
**EXERCISE**				
(23)	Meta-analysis	PTSD patients (*n* = 200)	Compared to control groups, exercise significantly reduced PTSD symptoms; Hedges' *g* = −0.35.	- Small number of studies (4). - High variety in exercise interventions used.
(24)	Meta-analysis	Healthy and obese participants and patients with either diabetes/ depression/MS/panic disorder (*n* = 1.111)	Single exercise session increases BDNF levels; Hedges' *g* = 0.46. Regular exercise intensified the effect of a session of exercise on BDNF levels; Hedges' *g* = 0.58.	- Several studies did not report on intensity of exercise.

Another issue of interest is the degree of realism the VR environment evokes. One can choose to project a VR environment which as realistically as possible resembles the original traumatic event, or one can choose to use symbolic representations within a virtual environment. The advantage of the primary option seems that a high level of realism will optimally support the retrieval of the traumatic memory. However, this has not yet been investigated and it has not yet been studied either whether increased realism leads to greater PTSD symptom reduction. Also, idiosyncrasy poses a problem, as it is a costly operation to develop a tailor-made environment to fit each patient. To tackle this problem, VR systems have been developed for veterans in which a clinician can add certain elements (such as a wounded soldier, gunfire, explosions) and modify certain settings (such as the ambient lighting) to personalize an environment to the patients' needs (17). Although elegant, this approach may be less viable for non-military populations. Another way to address this issue has been described by Baños et al. (25) and consisted of a generic environment which can be adapted with symbolic representations to reflect patients' emotional states. For instance, patients can select a threatening forest which is related to anxiety or a snowy town which can be related to a sad situation and place symbols in these environments that help them to retrieve and process these emotions.

Reviewing these options, for the TR-PTSD population it is of interest whether combining realistic virtual environments with symbolic representations would lead to an optimally supportive environment for retrieval of traumatic memories. An immersive VR environment in which the patient and therapist are together would heighten in-session attention without the risk of isolating a patient from the outer world. Next, including symbols selected by the patient could increase treatment engagement as patients are invited to co-create the virtual environment. In this way the idiosyncrasy issue is tackled by providing a generic environment that is personalized with symbolic representations that directly relate to the traumatic memories. We hypothesize this to hold great promise since this would lead to increased presence and engagement, lower avoidance and thus improved memory retrieval.

### Using multi-sensory input in therapy

#### Visual input; use of pictures or memorabilia

Retrieval of memories is often supported by administering multi-sensory input. On a visual level it has been found that viewing affective pictures leads to a specific physiological response pattern which is hypothesized to represent emotional engagement (3). Bradley et al. (3) also found this response to be higher when pictures are unpleasant as compared to pleasant. Using affective pictures to increase engagement and overcome avoidance during PTSD treatment is an easy and often applied technique used by clinicians during evidence-based exposure therapies (26). For instance, in Brief Eclectic Psychotherapy for PTSD [BEPP; (27)], bringing memorabilia is a standard part of the treatment protocol. In this treatment, patients bring pictures or tangible objects that remind them of the traumatic experience with the goal to promote engagement in exposure. Often, clinicians will use a similar strategy in EMDR or PE.

#### Auditory input; use of music

On an auditory level, music has proven to be a strong trigger for emotional biographical memories (28), even in persons with memory impairments (29). Listening to music evokes vivid and emotional memories in an even stronger way than looking at emotional faces (30). In patients with dementia, music selected by a researcher provoked autobiographical memory recollection (31). When patients themselves selected music, significantly more autobiographical memories were retrieved. A personal selection of music thus seems to be important for optimal memory recollection in patients with dementia and this may also be true for other disorders. Furthermore, studies with PTSD patients have shown that patients report music to evoke (traumatic) memories, which provides opportunities to access and discuss those memories (32, 33).

#### Role of olfactory input; odorants

Odors can quickly and strongly elicit autobiographical memories and associated affect (34). Compared to memories cued by auditory and visual information, these memories are more emotionally laden, which is known as the Proustian phenomenon (35). This could be explained by the relatively short (two synapses only) and unique connection between the olfactory bulb and limbic system with the amygdala (36). In PTSD patients, odors can evoke traumatic memories and even olfactory flashbacks (37). Because of these properties, olfactory cues could have the ability to enhance presence and probe traumatic memory recall during PTSD treatment. However, this is rarely utilized in practice (38, 39). To date only one study included olfaction as a variable in exposure therapy (VRET), but the contribution of this specific component is unknown (40). Before using odors as cues in treatment more should be known about its effects as its strong properties may possibly also result in unwanted results, such as higher levels of dissociation.

This literature points to the fact that pictures, music and odors can strongly elicit autobiographical memories, and therefore have the potential to enhance therapeutic processes of imaginal exposure in PTSD. Even music that is not selected by the person itself will lead to an affective response, and in a similar manner, non-personal affective pictures increase engagement. Both responses seem to increase when individuals use personal material or select it themselves. We therefore propose that it appears advantageous to self-select multi-sensory input during treatment to increase engagement and support activation of traumatic memories. In relation to a generic VR system with idiosyncratic representations as mentioned in the previous paragraph, multi-sensory input in the form of pictures and music could function as the described symbolic representation. Once more is known on the specific effects of utilizing odors in treatments, it should be considered to use these in treatment of PTSD as well.

### Movement as game-changer in therapy

#### Physical activity and exercise

Traditionally psychotherapy has been delivered in a sedentary position, however approaches in which patients can take a more active attitude are evolving. Exercise and physical activity interventions are being explored in the treatment of PTSD, either as a stand-alone treatment, as an adjunct to treatment, or as an augmentation to treatment. Physical activity is defined as “any bodily movement produced by skeletal muscles that requires energy expenditure” and exercise as “physical activity that is planned, structured, repetitive and purposive in the sense that improvement or maintenance of physical fitness or health is an objective” (41). Taken together these describe a wide range of activities, which is reflected in a recent meta-analysis on bodily movement interventions for PTSD (23). This meta-analysis included 4 randomized controlled trials: two trials administered yoga as stand-alone intervention (42, 43), one trial used a low-intensity combined aerobic and resistance training intervention as adjunct to care (44), and one trial used aerobic exercise (stationary cycling) directly before PE [augmentation to care; 45)]. See Table 1 for an overview of sample investigated, effect sizes, and potential confounders. Despite the small number of studies included in this analysis and the different interventions used, results are promising as a significant decrease of PTSD symptoms was found overall. However, the mechanisms thought to be involved in these interventions are quite different. Although yoga fits within the definition of exercise, its focus is more on flexibility than aerobic fitness. Yoga as treatment for PTSD aims to increase body awareness and emotion regulation, thereby providing patients with new coping mechanisms. In contrast, the exercise program administered by Rosenbaum et al. (44) is based on the premise that a more active and healthy lifestyle will result in improved (mental) health. The exercise program used in this study consisted of one supervised and two unsupervised 30-min resistance trainings per week, plus a pedometer and the encouragement to take 10,000 steps per day. The attendance rate of especially the unsupervised sessions was low, which is in line with research on adherence to exercise programs (46). However, the time patients spent walking increased from on average 285–412 min and PTSD symptoms of patients in the intervention group as compared to the usual care did decrease significantly. We hypothesize that the increase in walking is the main cause of this effect.

The meta-analysis also included a pilot controlled trial conducted by Powers et al. (45), in which the effects of stationary cycling as augmentation to exposure therapy were studied. Nine patients were allocated to either PE alone or PE proceeded by 20 min of moderate intensity exercise and results indicated a very large positive effect on PTSD symptoms in the PE plus exercise group as compared to PE alone. The working mechanism proposed by Powers et al. (45) for the effect of aerobic exercise as augmentation strategy to exposure therapy is based on elevating brain-derived neurotrophic factor (BDNF). BDNF is an essential factor for synaptic plasticity in the amygdala and hippocampus and plays a vital role in fear conditioning and extinction. In mice it has been shown that decreased BDNF release was associated with impaired fear extinction consolidation (47, 48), which could implicate that elevating BDNF in humans before, during, or after exposure might be beneficial for consolidation of fear extinction. In line with this work, Powers et al. (45) found higher BDNF levels in the patients who exercised before exposure in comparison to the patients who underwent exposure alone. Most studies achieved a raise in BDNF levels in the brain following moderate-intensity exercise, such as cycling or brisk walking (24, 49). One study applied normal walking as exercise intervention and also found increased BDNF levels (50). It is worth to note that increased BDNF in response to exercise is one possible working mechanism; alternative explanations for the effect of exercise in combination with exposure could for instance be the release of endogenous opioids, effects on NDMA receptors or effects on the HPA axis.

#### Physical activity and cognitive function

Physical activity could augment treatment via non-somatic pathways as well. A large meta-analysis showed that both high, moderate, and low-intensity exercise before a cognitive task have a positive effect on overall cognitive functioning and that cognitive performance on tasks that are performed during or directly after walking is also increased (51). Cognitive functioning has consistently been found to be impaired in patients with PTSD, most profoundly in the domains of verbal memory, learning, working memory, and speed of information processing [Overall effect size Cohen's *d* = −0.49; 52)] and these deficits can hinder treatment outcome (52, 53). Exercise could therefore augment treatment indirectly by improving cognitive functioning during treatment. Along the same lines, a study by Oppezzo et al. (54) examined the effects of walking on a treadmill as compared to sitting on separate tests for divergent and convergent thinking in two experiments. First, healthy participants (*N* = 42) performed both tests while sitting (sit), and then performed parallel tests while walking on a treadmill (walk). In the second experiment the initial condition was repeated and a sit-sit condition and a walk-sit condition were added. Both experiments found walking to specifically increase expression of associative memory (divergent thinking), which was measured with a test in which participants had to find alternative and original uses for an object. The amount of correct original suggestions doubled in all walking conditions (effect size first experiment: Cohen's *d* = 0.70). Divergent thinking is at the base of novel ideas, creativity and the free flow of ideas. Increasing divergent thinking in treatment of PTSD could result in increased expression of associative memory, and could therefore be a potential approach to decrease avoidant strategies. Interestingly, the researchers also studied the difference between inside walking (on a treadmill while facing a wall) and outside walking (in a peaceful forest) and found no difference between these conditions. This suggests that walking indoors during a therapy session could sufficiently promote divergent thinking. These effects seem to be specific to walking and not to exercise in general, as a study with a similar paradigm found aerobic exercise to actually decrease divergent thinking (55). Several authors have suggested that walking seems to have a specific combination of characteristics that distinguish it from other types of (low-intensity) exercise such as running or cycling (56, 57), however hard evidence for this hypothesis is not yet available.

#### Embodied cognition

Embodied cognition is the concept of “how states of the body modify states of the mind” (58) and has been studied in a broad spectrum of research fields. For instance, it has been found that (unconsciously) leaning toward the left will make you more prone to select statements that belong to left-wing parties and vice versa. In the same sense it has been found that physically approaching a feared object will result in positive appraisal of that object (59–61). Based on this premise Wolitzky et al. (62) performed an experiment in which 88 patients with acrophobia (fear of heights) either performed a standard exposure intervention of climbing open stairs and going higher every time anxiety went down below a certain level, or standard exposure intervention augmented with oppositional actions such as actively approaching a ledge by running toward it. Exposure combined with oppositional actions led to better behavioral and self-report outcomes than standard exposure, relaxation, or a waitlist. This showed that approach behaviors toward feared cues can augment treatment response and are of specific interest to consider in overcoming avoidance during treatment of PTSD.

Reviewing the elements of movement in this paragraph we think walking emerges as the optimal option. Walking appears to have a wide range of specific properties that contributes to improved expression of associative memory and divergent thinking, both when tasks are administered during and after the exercise. However, and in contrast to this, higher intensity-exercise, such as running or cycling, might actually decrease divergent thinking. Also, although not elaborately studied in PTSD patients yet, some evidence exists that walking contributes to an increase in BDNF levels, supporting fear extinction consolidation. Combined with knowledge on embodied cognition and approach behaviors, walking toward feared objects could provide the aforementioned benefits while simultaneously decreasing avoidance of the feared object. Because of these combined potential benefits and the high feasibility for most patients, we would opt for walking as augmentation strategy to PTSD treatment.

## The 3MDR intervention

### Framework for 3MDR

**Figure 1:**
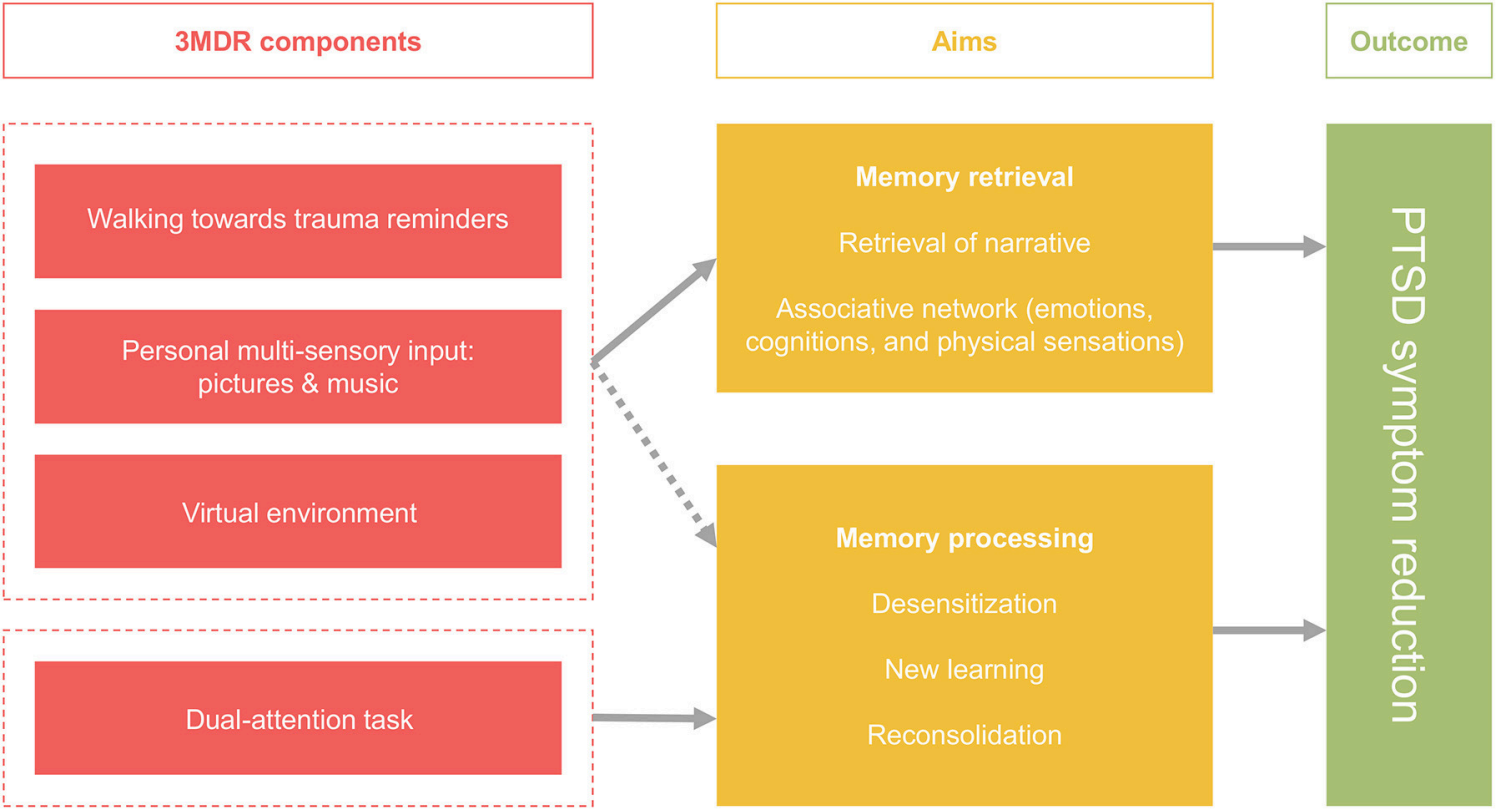
Schematic overview of the augmentation strategies applied in the framework of the 3MDR intervention and its outcomes.

Based on the strategies described above, a novel framework for treatment of patients with TR-PTSD has been developed: the Multi-modular Motion-assisted Memory Desensitization and Reconsolidation (3MDR) intervention. In this paradigm virtual reality, multi-sensory input, walking, and a dual-attention task are combined. The 3MDR intervention aims to promote memory retrieval and memory processing, ultimately resulting in PTSD symptom reduction. Memory retrieval is maximized by means of increasing engagement and overcoming avoidance. To this end patients walk toward trauma-related symbolic representations in the virtual environment. This action is hypothesized to have several results. Firstly, avoidance of the traumatic memory is expected to dissolve as patients walk toward the images that represent the memory, instead of (mentally) moving away from it. Secondly, being in the virtual 3MDR environment allows for the patient and therapist to be “present” in the virtual place and time, thereby increasing treatment engagement. Thirdly, multi-sensory input provides a direct and personal link to the traumatic memory and is therefore expected to break down avoidance in an even stronger way and promote memory retrieval and narration of this memory. Walking is expected to enhance this process because of its known effects on associative and creative thinking. In combination, these elements support retrieval of traumatic memories and associated networks of related memories, emotions, and cognitions. The second aim of the intervention is processing of the traumatic memories and networks through the process of exposure and by taxing the working memory with a dual-attention task. This strategy has been adapted from procedures as used in EMDR. Challenging the working memory during traumatic memory retrieval is expected to result in memory processing by means of new learning and reconsolidation of an adjusted memory trace. In the paragraphs below we will discuss the components and their roles in achieving memory retrieval and processing based on the literature review (for a schematic overview see Figure 1).

#### Walking

Walking toward the trauma-related pictures plays a crucial role in the 3MDR intervention. During a 3MDR session, patients walk on a treadmill at a brisk pace for on average 50 min. It activates patients both physically and mentally and thereby adds substantially to the aim of increased engagement and decreased avoidance. Based on the embodied cognition theories as described in section Embodied Cognition [e.g., (59, 62)] we expect the action of approaching an image that represents the traumatic memory to substantially decrease or even completely break down the tendency to avoid the traumatic memory. Furthermore, based on the work of Oppezzo et al. (54), walking is presumed to enhance divergent thinking, which facilitates accessing emotional and cognitive networks associated with the traumatic memory. This relates to the second aim of processing the traumatic memory as increased associative thinking could also allow for new (details of) memories to surface (54). Thereby, walking potentially supports the formation of a new cognitive and emotional experience related to the traumatic event, contributing to reconsolidation of the traumatic memory.

#### Virtual reality

As proposed in section The Promise of Virtual Reality, a generic VR environment has been designed to increase in-session attention and immersion [e.g., (17, 20)]. In contrast to VRET, the VR in 3MDR does not have the aim to realistically recreate the traumatic event(s). Instead, the main goal is to create an environment in which the patient can engage and can focus on moving toward trauma-related symbolic representations. To support this process the environment consists of tunnels at the end of which a picture is displayed with open spaces in between. In this way the VR environment allows for a focused and gradual exposure to the symbolic representation. Furthermore, using a generic environment, which is personalized with trauma-related multi-sensory input tackles the idiosyncracy issue. Together, therapist and patient are present in a CAVE, on which the virtual environment is projected. With the therapist by their side, patients interact with the self-selected pictures and music. This promotes emotional immersion as the therapist coaches the patient to approach the images, narrate the story of the traumatic events and address the traumatic hotspot (19).

#### Multi-sensory input

Multi-sensory input is delivered through symbolic representations, which consist of images and music that have been selected by the patient to strongly relate to the traumatic event. Images can range from pictures from a personal photo album, to drawings, pictures taken from objects or memorabilia, images from the internet or pictures from the environment in which the event took place. Music from the time of deployment is used as a symbolic representation of the entire period in which the traumatic events took place and a present-day neutral music is used as symbolic representation of the current time (to mark the end of every session). Based on studies as described in section Visual Input; Use of Pictures or Memorabilia the symbolic representations are expected to increase engagement and promote retrieval of the traumatic memories (3, 28, 30), thereby decreasing avoidance. Selecting these multi-sensory representations stimulates patients to start a creative associative process with regard to their traumatic memories and increases involvement as they co-create the therapeutic environment. Furthermore, as patients are asked to select pictures and music at home, it can contribute to addressing the experiences at home and having conversations about it with other family members, increasing engagement in treatment from the start. Despite the strong properties of olfaction as probe for traumatic memory retrieval, the current intervention does not incorporate an olfactory cue as the effects need to be studied systematically before doing so.

#### Dual-attention task

A dual-attention task is presented after narration of the traumatic event. In this task patients are asked to follow a horizontally moving ball across the screen with their eyes and call out numbers aloud that are displayed on this ball. The working mechanism of the dual-attention task used in EMDR (most often eye movements, which bears similarities with the task applied in 3MDR) has recently been argued to rely on reconsolidation and the learning of new information which can be added to the original memory trace (63). Several studies (64, 65) have provided support for this theory as they found that reconsolidation can indeed be disrupted in an adaptive manner by a distractor stimulus or task. This stimulus is thought to replace the representation of the conditioned (fear) response to the traumatic memory trace in the working memory, resulting in lower emotional valence of the memory. In 3MDR a dual-attention task is employed to facilitate reconsolidation as well. However, in contrast to in EMDR, during one session several picture cues (up to seven) are used to address several traumatic experiences. After each memory retrieval, the dual-attention task is administered once.

Reconsolidation was first described by Nader et al. (66) and has since then been established both in humans and animals. It is the process of memories becoming labile and malleable after activation, which is followed by a protein-synthesis dependent restabilization period in which reconsolidation of the memory needs to occur for it to persist. During the reconsolidation window (up to 6 h after retrieval) this process can be disturbed and memories can be stored in an altered form. It is hypothesized that PTSD symptom change follows after reconsolidation in combination with new learning during the reconsolidation window (67). Reconsolidation should occur directly during the distractor task after successful reactivation and therefore, repeated use of the task would be unnecessary (68). Often, veterans have experienced multiple traumas during one or more deployments and the associated memories, cognitive, and emotional associations are linked. Addressing hotspots from several traumatic memories within one session ensures activation of the full traumatic memory network and allows for new learning to occur as the patient is able to develop a more integrative narrative, which will not only enhance emotional immersion but also processing of the traumatic events. Additionally, patients' assumptions about themselves are challenged (e.g., changing from “*I am not able do this”* to “*I have overcome my fears”*), which is expected to result in a sense of empowerment.

In sum, we hypothesize that the above-mentioned components result in a psychotherapeutic approach in which (emotional) participation in treatment is increased. The full traumatic network is thought to be activated and intervening in the reconsolidation process in addition to new learning is expected to result in lower emotional valence and processing of the traumatic memories. In combining these components, the therapeutic setting changes drastically from a passive face-to-face position to an active side-by-side position. This not only has impact on the physical context, but also on the therapeutic alliance as the therapist is literally standing next to the patient, supporting him or her in the process. In a proof-of-concept study the intervention and first experiences of veterans with PTSD have been found to be promising (69).

### Outline of the 3MDR intervention

The 3MDR intervention consists of one to two preparatory sessions, six 3MDR sessions, and evaluative sessions. In the treatment of TR-PTSD patients it can be applied as a breakthrough therapy and if needed can be followed up with other types of (trauma-focused) psychotherapy. In the preparatory sessions, patients receive psycho-education and the assignment to select the symbolic representations that will be used in the 3MDR sessions. After careful explanation of this assignment patients select pictures and music at home. They bring these to the preparatory sessions and together with their therapist decide on which material is suited best and whether material is missing.

**Figure 2:**
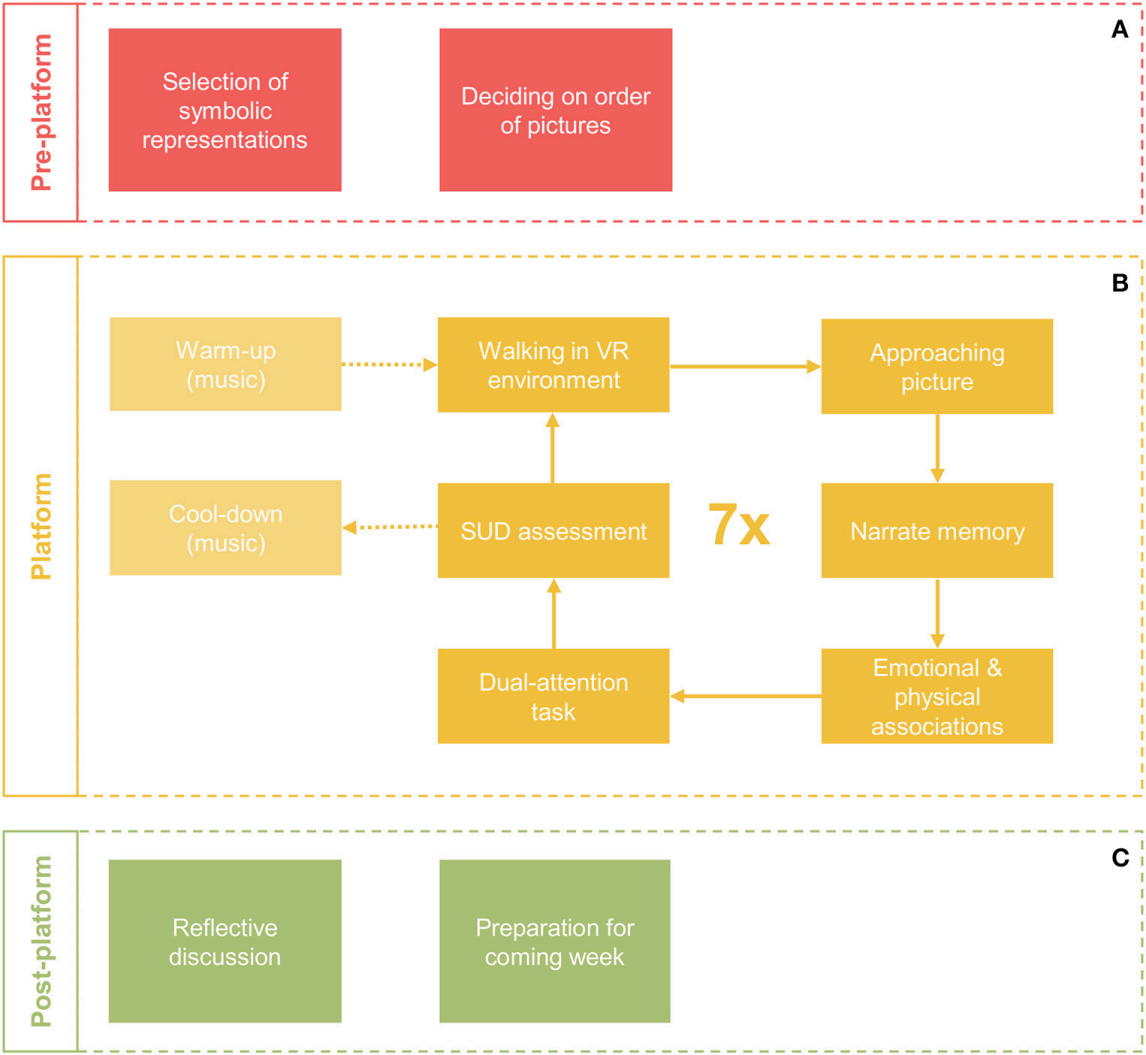
Schematic overview of a single 3MDR session. A session consists of a pre-platform phase **(A)**, a platform phase **(B)** and a post-platform phase **(C)**.

The 3MDR sessions consist of three phases: *pre-platform, platform*, and *post-platform*. For a schematic overview, see Figure 2. The current paper focuses predominantly on the platform-phase which is performed within the framework described previously. However, the other two phases are of no less importance in the therapeutic process. During the pre-platform phase, therapist and patient select and decide on the pictures that will be used during the session. Topics such as avoidance to selecting pictures will be discussed and patients will be guided to contribute to the selection of this material. In the post-platform phase, patient and therapist, take time to reflect on the session and focus on important or newly arisen cognitive and emotional associations and integrate this in the patient's daily life situation.

The platform-phase of every 3MDR session starts with a mental and physical warm-up during which the patient walks on the treadmill in a neutral virtual environment and music from the time of deployment is played. After this song, a cycle of ~5 min is repeated seven times with seven different pictures, which are highly affective for the patient. During this cycle, the patient will walk toward a picture and the therapist, who is standing next to the patient, will support the patient to narrate about the picture when this is maximally displayed on the screen (e.g., “*what do you see in this picture?”*), the traumatic event (e.g., “*can you tell me what happened?”*) and the associated emotional and physical associations that the patient is feeling at that moment (e.g., “*what feelings do you experience now?*” or “*where do you feel this in your body?”*). To capture these associations they will be typed out by the session operator and projected on the screen, superimposed on the picture (e.g., “*powerless”* or “*I feel butterflies in my stomach”*). After the narration is completed the dual-attention task follows. This is followed by rating of the subjective units of distress by the patient. After seven cycles, a mental cool-down follows during which present-day song is played and the patient is invited to leave the memories behind and return to the “here and now.” The patient will walk during the entire platform phase, on average covering a distance of 3–4 km during one session.

The duration of a typical 3MDR session is 90 min, of which a patient walks on the platform for 45–60 min. This timeframe is in accordance with other trauma-focused therapies (27, 63) and allows for addressing 7 pictures. In the first proof of concept study (69) patients received 4 sessions of 3MDR. The patients reported that they would have liked to receive more sessions. In the next phase we therefore experimented with 6–8 sessions, resulting in the decision of 6 sessions as the optimal number for patients to achieve the boosting effect of the treatment. In TR-PTSD patients, the 3MDR treatment is used as a breakthrough intervention and is followed by other treatment if needed. Ongoing systematic evaluation of the 3MDR treatment will show whether the amount of sessions is accurate or needs to be adjusted in the future.

### Illustrating the application of 3MDR: case examples

To illustrate the protocol of the 3MDR intervention we present three case examples of veterans with TR-PTSD who received between 4 and 6 sessions of 3MDR at Foundation Centrum'45. Next to a description of the patients' experiences we report on PTSD symptom severity pre- and posttreatment as measured with the PTSD Checklist for DSM-5 [PCL-5; (70)], which is part of routine outcome assessments at Foundation Centrum '45. This study was carried out in accordance with the recommendations of the Medical-Ethical Review Committee (METC) of University Medical Center Utrecht, with written informed consent from all subjects. All subjects gave written informed consent in accordance with the Declaration of Helsinki. The protocol was approved by the Medical-Ethical Review Committee (METC) of University Medical Center of Utrecht. Additionally, the participants gave consent for the description of their case as presented below. To protect the privacy and identity of the participants, their names have been changed.

#### Case example luke

Luke is a veteran who had been deployed for the French foreign legion. He suffered from severe TR-PTSD, with nightmares, feeling hyper-alert, and avoiding public places as his most prominent symptoms. He had been in treatment for 3 years during which he received several treatments, including EMDR and medication over the course of 2 years, without sufficient symptom relief. He described that he had difficulty with focusing on one memory (hotspot) during EMDR and that other hotspots would pop up and distract him.

As Luke did not have pictures from his time of deployment, he looked for images online. Although this proved to be a tough and demanding task, he succeeded in finding pictures that addressed his memories adequately. He reported that walking during the 3MDR was important as he had a real sense of walking back in time, especially when combined with his music. During the sessions he experienced intense emotions, which up until now he has been able to suppress. In previous treatment settings he could feel those emotions surface but was always able to keep them inside. During 3MDR he felt he could allow his feelings to be there. Also, he felt as if all the different traumatic experiences were linked in his mind but that those links were blocked and now opened up.

Luke experienced a positive effect of the 3MDR sessions a few weeks after the last session. He noticed that it was easier to talk about his experiences and that he started to remember positive events from his time of deployment. He felt less alert, his nightmares disappeared, and he was now able to go to busy public spaces without scanning for potential danger or feeling anxious in a crowd. This was reflected on the PCL-5, as his score decreased with 25 points, which indicated clinical significant change and he no longer met criteria for a PTSD diagnosis posttreatment. At 6-month follow-up his score on the PCL-5 dropped to 6.

#### Case example nick

Nick is a veteran who had been deployed to Lebanon. His PTSD symptoms had developed progressively, with severe aggressive behavior, startle reactions, flashbacks, difficulty sleeping, and difficulty to talk about his experiences in Lebanon. He had received non-trauma-focused treatment and several pharmacological treatments for a total of 6 years without symptom relief.

Nick described that during the first session he felt as if a deep wound had been scratched open, which deepened over the course of his four 3MDR sessions. He experienced intense emotions such as sadness and anger, and was surprised to notice how these emotions changed. At several moments he cried, which gave a sense of relief to him. Nick valued the use of the pictures and music and with this support had no trouble retrieving his traumatic memories, which had been hard for him prior to 3MDR. He reported that during the 3MDR the full memory of his time of deployment was activated. Despite the realistic experience, he felt completely in control.

Nick reports to have benefitted from the 3MDR treatment in that he experienced a breakthrough in his emotions. Also, he remembered new elements of the time of deployment. Although he stated that this was very important to him, this positive effect was not reflected on the PCL-5, as his PTSD symptom severity score increased slightly with 8 points, which does not reflect a clinical significant change. His progress was complicated because shortly after his last 3MDR session Nick's wife passed away.

#### Case example peter

Peter is a veteran who had been deployed to Afghanistan. He experienced severe TR-PTSD symptoms, including a strong withdrawal from society to avoid all potential triggers. He had been treated with EMDR by an experienced therapist for 6 months, however this did not result in any symptom relief. During the EMDR he would be easily distracted, and was not able to stay focused on the memory and associative cognitions and emotions.

The first two 3MDR sessions Peter found very intense and confronting, and he experienced some symptom aggravation, which dissolved in the following sessions. Walking was important for him as he felt more comfortable in this active position and it helped him to really focus on the memory without getting distracted. In processing of his memories, Peter emphasized the importance of the talk directly after 3MDR during which he and his therapist shortly reflected on what had come up and he received suggestions on how to deal with these things at home.

Peter said he has opened up to life again. He no longer avoided work, going to busy places or doing sports. Also, he noticed improvement in parenthood; he expressed to be able to function again as the dad that he wanted to be. Furthermore, he remembered positive aspects of his deployment to Afghanistan and felt proud of what they achieved during their time there. These positive effects were reflected in a drop of 30 points on the PCL-5, which is a clinical significant change and he no longer met criteria for a PTSD diagnosis.

## 3MDR in the context of other innovations in TR-PTSD treatment

Several other innovative treatments are currently being considered and show potential for treatment of TR-PTSD as well. Without aiming to be exhaustive, various directions can be distinguished, such as an intensification of treatment, using pharmacological agents to augment treatment (with for instance MDMA or propranolol), and using technical devices in treatment (such as neuromodulation techniques). Below we will discuss 3MDR in the context of intensifying treatment and reconsolidation interventions, as these development are most closely related to the 3MDR approach.

### Intensifying treatment

Several intensive treatment protocols have been developed in which patients receive a trauma-focused therapy (twice) daily during 1–2 weeks. Ehlers et al. (71) found intensive cognitive therapy to be as efficacious as normal cognitive therapy, thus achieving the same symptom reduction in a shorter period of time. In a treatment study with veterans a VR based exposure treatment was delivered as part of an intensive outpatient treatment program of 3 weeks, after which 66% of the patients treated no longer met criteria for PTSD (72). Case-reports with VRET (73), EMDR (74), and PE (75) as intensive trauma-therapy have been described as well. It is hypothesized that such intensive short-term treatment formats will diminish between-session avoidance and prevent drop-out next to helping patients to keep the therapeutic material more active in their mind. The 3MDR has a similar yet clearly distinctive aim to decrease within-session avoidance by intensifying therapy with a three-fold augmentation strategy in single sessions. It is of interest whether the intensive treatment formats could provide relief for TR-PTSD patients by for instance actively trying to prevent drop-out, however this has not yet been studied for this population specifically or in comparison to standard evidence-based treatments.

### Reconsolidation interventions

Another intervention that is potentially relevant for TR-PTSD concerns intervening in reconsolidation as therapeutic mechanism. Pitman et al. (76) were among the first to conduct a pilot controlled trial into the reconsolidation blocking effects of propranolol. They hypothesized that administration of this pharmacological agent after memory retrieval blocks protein synthesis necessary for reconsolidation, thereby weakening the memory. They found patients treated in this manner to show significantly greater PTSD symptom reduction as compared to a placebo control group. Patients in this sample however, had low clinician-rated PTSD scores at the beginning of the trial already, and were not representative for the TR-PTSD population. Kindt et al. (68) performed 4 case studies with a similar protocol and included one patient with TR-PTSD. After the propranolol intervention, this patient no longer met criteria for PTSD. However, another patient in this study did not respond, which was explained by difficulties with retrieval and activation of the traumatic memory trace. This underlines the importance of providing sufficient cues for successful reactivation, after which altered reconsolidation can occur. Several studies have reviewed other strategies to intervene with reconsolidation, such as performing a cognitive dual-attention task (64, 65), or performing physical exercise prior to exposure (45) as both applied in 3MDR. An advantage of these alternative forms of interruption over propranolol is the non-invasive character of these strategies. However, less is known about the exact working mechanisms for these interventions as compared to those of propranolol. Research supporting that these strategies actually interact with reconsolidation processes is steadily growing (64).

## Concluding remarks and future directions

Driven by the need to improve treatment for patients with TR-PTSD we explored ingredients for an innovative framework for delivering trauma-focused psychotherapy, called 3MDR. We showed how an intensive combination of state-of-the-art technology, personal multi-sensory input, and walking is expected to decrease avoidance and increase engagement, resulting in improved memory retrieval. We emphasize the importance of walking as augmentation strategy as it is hypothesized to result in increased associative thinking, overall activation and fear extinction consolidation. Moreover, walking toward a feared object would result in positive appraisal of this object, thereby providing a potential method to decrease the natural tendency to avoid traumatic memories. Simultaneously, processing of the traumatic memories and associated affect through desensitization, new learning and reconsolidation is optimized by applying a dual-attention task. The case examples illustrated these mechanisms of action as the patients reported how walking supported breaking through avoidance, and remaining focused, and how the use of VR and multi-sensory input made it easier for them to engage in treatment, retrieve the traumatic memories, and to process the emotions connected to the trauma. Furthermore, they recognized the importance of addressing several traumatic memories in one session and the talk with the therapist after each session, which helped them to process their traumatic memories.

Looking at the different approaches in the field to improve care for patients who are suffering from TR-PTSD, strategies focusing on intensive short-term treatment formats and optimizing reconsolidation, are related to strategies used in 3MDR. What distinguishes 3MDR from these approaches is that high avoidance and low engagement are viewed as target factors for increasing treatment outcome. However, further research should show whether this assumption is appropriate by identifying profiling factors for patients with TR-PTSD as determinants for their course of treatment. Aided by consistently proven profiling factors, treatment can be adequately personalized to a patient's needs in the future. Recently a staging approach to PTSD was proposed (77) in which progressive stages of severity of the disorder are associated with specific (bio)markers. Further development of such a model should allow for grounded claims on which intervention to use during which phase of the disorder (78).

Some TR-PTSD patients might have shown a non-response to treatment by for instance responding to trauma-focused treatment with severe emotional dysregulation, like strong dissociative reactions or psychotic symptoms. Because of the strong immersive nature of the 3MDR treatment, these types of responses could potentially be a contra-indication to 3MDR. As in other trauma-focused therapies, comorbid severe depression with limited affect modulation and personality disorders that prevent the formation of a therapeutic alliance could also negatively interfere with the therapeutic process. Additionally, the strong immersive capacities of the VR could also result in an aversive reaction in which avoidance is increased, potentially resulting in poorer treatment outcome. It is important that well-trained and supervised therapists pay attention to the therapeutic relationship and keep a close eye on adequate graded exposure to traumatic events in patients who are prone to react with strong dysregulations. Future research is expected to shed more light on actual contra-indications and nature of aversive reactions.

In conclusion, based on an evaluation of augmentation strategies, in this paper we propose an innovative framework for psychotherapy of treatment-resistant PTSD. Three case examples serve as illustration of this approach. Combining therapeutic principles in a virtual-reality, multi-sensory, motion-assisted, intensive paradigm has the potential to drastically change the way in which therapy is delivered, being from sedentary and face-to-face to an active and side-by-side context. The focus on increasing treatment engagement and overcoming avoidance in patients that do not benefit sufficiently from other therapies could possibly improve treatment outcome and adherence for this group. We acknowledge that the evidence for this treatment approach is still limited and based on clinical case reports described in current and earlier work (69). For now, we are cautiously enthusiastic on this framework for PTSD treatment and encourage a rigorous evaluation of the efficacy of 3MDR. Randomized controlled trials for patients with TR-PTSD are currently being performed. Future research will provide insight into whether the 3MDR approach is efficacious for PTSD patient populations that have shown to benefit less from evidence based treatments, whether 3MDR is to be applied as a first line treatment, and whether non-military PTSD patients populations could benefit from this treatment as well. This novel framework can serve as a base for development toward further personalization of psychotherapy and interactive approaches to treatment.

## Author contributions

MvG, MN, and EV have provided substantial contributions to the conception or of the work and the interpretation of the data. Additionally MvG has had a substantial contribution in the acquisition of the data for the work. MvG, MN, and EV have made a substantial contribution in drafting the work and revising it critically for important intellectual content. MvG, MN, and EV all provided final approval of the version to be published and agree to be accountable for all aspects of the work in ensuring that questions related to the accuracy or integrity of any part of the work are appropriately investigated and resolved.

### Conflict of interest statement

The authors declare that the research was conducted in the absence of any commercial or financial relationships that could be construed as a potential conflict of interest.
